# Perspectives on contraceptive implant use in women living with HIV in Cape Town, South Africa: a qualitative study among primary healthcare providers and stakeholders

**DOI:** 10.1186/s12889-019-7312-1

**Published:** 2019-07-26

**Authors:** Anna Brown, Jane Harries, Diane Cooper, Chelsea Morroni

**Affiliations:** 1 School of Public Health and Family Medicine, University of Cape Town, Cape Town, South Africa; 2 Women’s Health Research Unit, School of Public Health and Family Medicine, University of Cape Town, Cape Town, South Africa; 3 School of Public Health, University of the Western Cape, Cape Town, South Africa; 4 Department of International Public Health, Liverpool School of Tropical Medicine, Liverpool, UK; 5 Botswana UPenn Partnership, Gaborone, Botswana; 6 Wits Reproductive Health and HIV Institute, University of the Witwatersrand, Johannesburg, South Africa

**Keywords:** HIV, Antiretroviral therapy, Contraceptive implants, Long-acting reversible contraceptives, LARCs, South Africa, Qualitative

## Abstract

**Background:**

This study explored primary healthcare provider and HIV/contraception expert stakeholder perspectives on South Africa’s public sector provision of contraceptive implants to women living with HIV. We investigated the contraceptive service-impact of official advice against provision of implants to women using the HIV antiretroviral drug, efavirenz, issued by the South African National Department of Health (NDoH) in 2014.

**Methods:**

Qualitative data was collected in Cape Town in 2017 from primary healthcare contraceptive providers in four clinics that provide implants, as well as from other expert stakeholders selected for expertise in HIV and/or contraception. In-depth interviews and a group discussion explored South Africa’s implant introduction and implant provision to women living with HIV. Data was analysed using an inductive thematic analysis approach.

**Results:**

Interviews were conducted with 10 providers and 10 stakeholders. None of the four clinics where the providers worked currently offered the implant to women living with HIV. Stakeholders confirmed that this was consistent with patterns of implant provision at primary healthcare facilities across Cape Town. Factors contributing to providers’ decisions to suspend provision of the implant to women living with HIV included: inadequate initial and ongoing provider training; interpretation of NDoH communications about implant use with efavirenz; provider unwillingness to risk harming clients and concerns about professional liability; and other pressures related to provider capacity.

**Conclusions:**

All South African women, including those living with HIV, should have access to the full range of contraceptive options for which they are medically eligible. Changing guidance should be initiated and communicated in consultation with primary-level providers and service beneficiaries. Guidance issued to providers needs to be clear and fully evidence-informed, and its correct interpretation and implementation facilitated and monitored. Guidance should be accompanied by provider training, as well as counselling messages and tools to support providers. Generalized retraining of providers in rights-based, client-centred family planning, and in particular implant provision for women with HIV, is needed. These recommendations accord with the right of women living with HIV to access the highest possible standard of sexual and reproductive healthcare, including informed contraceptive choice and access to the contraceptive implant.

## Background

Women’s informed and voluntary use of effective contraception reduces their risk of negative health and socioeconomic outcomes associated with unintended pregnancy [1, 2]. Offering all women a range of contraceptive options is essential to their sexual and reproductive health rights and heightens their ability to make informed, personal choices which, in turn, increases contraceptive uptake, acceptability and effective use [3–5]. For women living with HIV, improving contraceptive choice and service quality may have added benefits of enabling pregnancy planning. This has been shown to be an important strategy for reducing HIV-associated maternal and child morbidity and mortality, and for preventing vertical HIV transmission [6–8].

Efforts to better meet the contraceptive needs of women in South Africa, including of the 21–23% of reproductive-aged women estimated to be living with HIV [9–11], were made in 2012 through a comprehensive revision of the 2001 national contraceptive policy. Developed by the South African National Department of Health (NDoH), the National Contraception and Fertility Planning Policy and Service Delivery Guidelines [12] and the accompanying National Contraception Clinical Guidelines [13] were designed to expand contraceptive method choice and access, and to align South Africa’s guidance with the World Health Organization’s (WHO) Medical Eligibility Criteria (MEC) for contraceptive use [14]. Expanding access to long-acting reversible contraceptives (LARCs) (i.e., intrauterine contraception and sub-dermal contraceptive implants) was central to these revisions. LARCs require no action for continued use on the part of users once in situ and have extended durations of use. This makes them convenient to use and highly effective [12, 13, 15]. In this context, the etonogestrel contraceptive implant was introduced into South Africa’s public sector contraceptive method mix in 2014 [16, 17].

Implant uptake in South Africa was initially promising but has declined since introduction in 2014 [16], with concerns about side effects and inadequate health care provider training as identified contributors underlying poor provider and community perceptions of implants [16, 18, 19]. Further, an NDoH circular to healthcare providers in late 2014 advised against implant provision to users of certain enzyme-inducing drugs, including the HIV antiretroviral drug, efavirenz (a key drug used in the country’s first-line antiretroviral therapy (ART) regimen) [20]. Citing pharmacokinetic [21] and some clinical data [22] showing that implant hormone levels are reduced in the presence of efavirenz, potentially reducing contraceptive effectiveness, the circular strongly advised against implant use and recommended implant discontinuation among implant users also using efavirenz [20]. This circular was followed months later by a brief stating women using these medications may choose to keep implants if already in situ, provided they were ‘*properly counselled about the increased risk of pregnancy because of drug interactions’* and about ‘*the importance of using additional non-hormonal contraceptive measures’* [23]. However, no supportive training or counselling resources accompanied this advice. The WHO MEC states in respect to use of the implant with efavirenz that ‘*the advantages of using the method generally outweigh the theoretical or proven risks’* [24]. The initial circular was controversial because it did not align with this WHO MEC guidance. Moreover, the 2014 NDoH recommendations did not recognize and communicate the importance of potential users’ needs, rights and preferences when making contraceptive decisions, which was deliberately emphasised in the 2012 revisions to national contraceptive guidance and is an essential aspect of rights-based, client-centred, high-quality contraceptive care as outlined by the WHO [5, 12, 13]. Subsequent research published in 2015 showed that despite some level of decreased implant effectiveness among women using efavirenz-based ART, the rate of unintended pregnancy remains much lower for these women than for women not using contraception [25]. Women using the implant while taking efavirenz-based ART are also at a lower risk of experiencing unintended pregnancy than women on efavirenz who use other methods that require regular adherence for effective use, such as injectables and pills [25, 26].

While the appropriateness of offering implants to women living with HIV on efavirenz-based ART has been explored [27, 28], there has been no assessment of the impact of official guidance on this topic on implant provision in South African primary healthcare settings. The objective of this study is to explore South African primary healthcare providers’ perceptions, knowledge and practice concerning implant provision in the context of HIV and ART. Primary healthcare providers’ perspectives are contextualised by those of policy, academic and clinical stakeholder experts working in HIV and/or contraceptive care. This research has relevance for countries and HIV/contraception programmes providing the contraceptive implant in settings of high HIV prevalence.

## Methods

### Study design, participants and recruitment

This study presents qualitative data collected in semi-structured, in-depth individual interviews and one group discussion with public-sector primary healthcare contraceptive providers and other expert stakeholders (specialist contraception providers; policy-maker/programme managers and academics working in HIV and contraceptive programming, training, clinical care, and research).

The primary healthcare providers (hereafter referred to as providers) interviewed include all of the providers providing family planning services at four public-sector primary healthcare clinics. Clinics sampled service a broad cross-section of communities in the Cape Town area and are situated in a variety of low-income urban and peri-urban areas. Women seeking contraceptive services at these clinics are from diverse South African and migrant backgrounds and of various reproductive ages, including adolescents and young adults. All four clinics provide contraceptive implants on site in addition to a range of other contraceptive methods (injectables, oral pills, condoms, and intrauterine contraception). Three of the clinics offer generalised primary healthcare services and one specialized in adolescent services. Three provide HIV diagnosis and treatment; one refers HIV-positive clients for HIV treatment services after diagnosis. Expert stakeholders (hereafter referred to as stakeholders) were purposively selected based on their roles and knowledge from a list compiled a priori by the researchers who had knowledge of key stakeholders in the Cape Town area.

### Data collection

Interviews were conducted between May and August 2017. Providers were given an information flyer about the study and invited to be interviewed with the assistance of clinic managers. Stakeholders were contacted via email, informed about the study and invited to participate. Separate interview guides were developed for providers and stakeholders. Prior to finalisation, the guides were piloted for flow and clarity with members of the research team as well as representatives from each of the two participant groups who were not included in study sample. Participants in both groups were asked about their overall perspectives on the implant, its introduction and its provision in the context of HIV and ART. Provider interviews focused on knowledge, understandings and interpretations of official guidance about the implant and efavirenz and its impact on their own practice, and that of colleagues. Stakeholder interviews focused on the official guidance development process and the reception, interpretation, implementation and impact of the guidance at the primary healthcare level. Suggestions to improve contraceptive choice, care and service provision for women living with HIV in primary healthcare settings in South Africa were sought from all participants.

Interviews were conducted in a private space in English by the author (AB), a young, female, University of Cape Town Masters of Public Health student with an English-speaking background. They averaged 45 min duration. While most providers’ first language was isiXhosa or Afrikaans, English is the usual language adopted in the healthcare workspace in South Africa. All participants confirmed that they were comfortable with being interviewed in English by AB and indicated that no interpretation was required.

The majority of interviews with providers were conducted individually, except for a group interview at one clinic at the request of the clinic manager. This was due to time constraints and staff work schedules on the day of interview. The four provider participants in the group consented to a group format and the research team decided that accommodating the participants in this regard would not compromise the quality or interpretability of the data. All interviews with stakeholders were conducted individually at their places of work.

Provider interviews ceased once data saturation was reached (i.e., the same themes were coming up repeatedly). The stakeholder sample was predetermined by the key stakeholder list compiled by researchers a priori*.*

Interviews were audio recorded. Informed consent, including permission to record interviews, was obtained from all participants prior to interview. Audio files were deleted after completion of transcription.

### Data management and analysis

Author AB transcribed interviews verbatim and reviewed them alongside voice memos and field notes. Initial coding was conducted by AB, using NVivo Software. Two senior study team members with extensive qualitative experience (authors JH and CM) reviewed all transcripts, codes, and themes with AB, discussed and resolved differing opinions on coding and interpretations, and the final codes were agreed by the three study team members. Inductive thematic analysis was used to identify themes, and results were organized by the interview guides’ main domains: perceptions of South Africa’s implant introduction and experiences of provision; impact on practice of the NDoH implant-efavirenz guidance; influence of training, capacity and health systems issues on interpretation and implementation of implant-efavirenz guidance; and suggestions for future contraceptive policy-making and service delivery initiatives.

## Results

Ten providers, all professional nurses, trained in and currently providing family planning and contraception services, and 10 stakeholders were interviewed. Stakeholders were three government women’s health and HIV programme managers, two HIV clinical researchers, two obstetricians/gynaecologists (OB/GYNs), a specialist contraception provider, a family planning trainer, and a pharmacologist.

None of the clinics at which providers were working currently offered the contraceptive implant to women living with HIV, regardless of whether potential implant users were receiving efavirenz-based ART, other ART regimens, or no ART. At all clinics, implants were being offered to HIV-negative clients. Stakeholders confirmed that restrictions on implant provision to women living with HIV were common in clinics across the greater Cape Town area.

### Perceptions of the implant introduction and experiences of implant provision

Providers and stakeholders reported enthusiasm surrounding the implant’s introduction in 2014, as a welcome new method important to expanding contraceptive options, particularly LAR Coptions, for South African women. A family planning trainer, with several decades’ experience at an HIV-focused non-governmental organisation, stated, *'when [implants] came here…Wow! Here is something that is going to save [time], and which does not need the clients to come to the clinic very often. It was a saviour, in our eyes'* (family planning trainer, female).Providers likewise reported initial optimism about the implant, with one describing it as 'revolutionary when it came' (professional nurse, female). However, some stakeholders lamented the early portrayal of the implant as a wide-reaching, singular solution to South Africa’s high rates of unintended pregnancy: *'The method was sold to the public and to the staff as if this is going to be the solution for all the family planning woes that we’ve had until now. Which of course it isn’t'* (health manager, female). Stakeholders involved in the provider training during the initial implant introduction perceived the implant introduction process as *'rushed', 'target-driven', 'not well-coordinated',* and *'a poor reflection'* of the 2012 guidelines’ intentions to expand and respect women’s informed contraceptive choices: *'The implant was completely foreign to [providers], so I think the training on its provision was quite rapid and the focus was insertion - to train them how to put it in, but the training on how to counsel women about their options was lacking'* (OB/GYN, female). Another stakeholder involved in the national implant provider training programme acknowledged that, *‘whilst everyone denies it, I think there was very much acascading of ‘see one, do one, teach one"* (specialist contraception provider, female).

Confirming stakeholders’ perceptions of a rapid decline in implant uptake after initially high levels, providers reported that, while the implant had been very popular when it was first introduced, insertions at their clinics had declined quickly thereafter and continued to do so. Providers perceived that large numbers of clients requested their implants be removed soon after insertion. In these cases, providers reported feeling ill-equipped to manage women’s concerns about the method in an evidence-informed way, often being unsure of whether particular side effects reported by clients, such as irregular bleeding, weight changes, headaches, and hair loss, were likely to be linked to the implant and/or how to appropriately counsel on and manage these side effects. Providers also reported that their clients were concerned about information they had heard about the implant in their communities, and that they were ill-equipped to respond to this. Such information included that the implant may be a *‘chip’* used for surveillance of women, as well as reported incidents of gangs violently removing implants from women’s arms. Provider’s believed that this negative information about implants had circulated widely in communities from soon after introduction and deterred women from initiating or retaining the method. Several stakeholders corroborated that they had heard reports of this kind of information from providers and clients.

### Impact on provider practice of the NDoH implant-efavirenz guidance

Providers and stakeholders generally reported that the late 2014 implant-efavirenz guidance from the NDoH was received at the same time as the general enthusiasm about the implant’s introduction was diminishing among potential users and providers. Providers reported that during this period, in late 2014, clients were expressing growing and multidimensional concerns about the method, removal requests were increasing, and uptake had started to decline. This lead to general provider apprehension about suggesting or providing the implant to women seeking contraception.

Providers and stakeholders generally remarked that the 2014 NDoH circular which stated that “women who are on [efavirenz] should not use […] implants” [20] and the subsequent brief which used language related to “safety” as opposed to “contraceptive effectiveness” to describe the issue [23] intensified general provider apprehension about the implant method, and also introduced specific concerns about the safety of its use in women living with HIV. Most stakeholders believed that there was an inadequate consultation process prior to the issuing of the NDoH guidance. They mentioned several problems with the guidance that they believed were a result of inadequate consultation: that important differences between medical safety and contraceptive effectiveness were not clarified; that there was no mention of relative contraceptive effectiveness of different contraceptive methods; and that the guidance had not adequately acknowledged the importance of informed contraceptive method choice and the role of women’s varying individual needs, values and preferences when making decisions about which contraceptive method to use. Most stakeholders and providers viewed the guidance as having effectively removed the implant as an option for women on efavirenz-based ART, which then was applied at the primary healthcare level to all women living with HIV. They described the guidance variously as ‘*pre-emptive*’, ‘*dogmatic*’, ‘*rushed*’, ‘*lacking perspective [with respect to the fact that absolute contraceptive effectiveness is only one of several factors a woman considers when choosing a contraceptive and that later research has shown that other methods such as injectables and oral pills when used with efavirenz may have higher rates of contraceptive failure than the implant]*’. The guidance was described by the majority of those interviewed in both groups to have been worded such that providers might conclude from it that implants were *‘not medically safe, so let’s just remove them’* (OB/GYN, female).

All providers confirmed that they believed implants to be totally contraindicated in the context of HIV infection, and that this was the general interpretation and implementation of the NDoH guidance at their clinics and elsewhere. This confirmed stakeholders’ general perceptions of restricted provision of the implant to women living with HIV across Cape Town primary healthcare clinics. Illustrating this, a health manager believed that providers in her sub-district were reluctant to offer a method they perceived might harm clients, with concerns about side effects seen as a deterring factor: ‘*No one is being offered – no HIV-positive woman is being offered [implants]. I think they’re just playing safe’* (health programme manager, female).

Current levels of knowledge and understanding among providers as to the NDoH rationale behind the implant-efavirenz directives ranged from vague notions that the implant should never be provided to ‘*chronic clients [including those living with HIV]’* (professional nurse, female); to, *‘I’m not sure which one, exactly, the ARV that is contraindicated with this [implant] …*’ (professional nurse, female); to, ‘*We were told the ART, it lowers the effectiveness - we were told, but how? I never went through it, or understood it quite clearly’* (professional nurse, female)*;* to*, ‘They say, it’s contraindicated with the drugs that they’re using, and that person can still fall pregnant when they are using the [implant] because it is not stronger than the three-months injection’* (professional nurse, male)*.*

While some providers were aware of the WHO’s MEC recommendations on implant use with efavirenz, all were reluctant to provide implants to women living with HIV pending clear, updated instructions from the NDoH. Providers described feeling a sense of personal responsibility for client safety and personal vulnerability, as well as fears of legal repercussions. *‘It’s not that I’ll find it difficult to insert, but at the end of the day, I’ll be held accountable. Anything bad that may happen… Some may lay charges, you know?’* (professional nurse, male).

Stakeholders were more aware of the evidence surrounding the NDoH’s recommendations and viewed it in accordance with their own understandings of the risk and benefit decision-making key to providing implants in the context of efavirenz. They were also more aware than providers that the guidance was specific to efavirenz-based ART rather than to all ART regimens or all women living with HIV. Rather than denying the implant as an option altogether for women living with HIV on efavirenz-based ART regimens, specialist contraception providers generally endorsed the following approach with clients: explaining the increased risk of implant failure with efavirenz, the reasons for this, the relative effectiveness of other methods, and the importance of dual contraceptive method use with condoms in addition to the implant to increase effectiveness, Justifying this, they cited the WHO’s MEC, the original 2012 guidelines which emphasize the importance of giving all women choice (including women living with HIV), and evidence indicating higher comparative typical-use failure rates of most other available methods. One specialist contraception provider had written letters for her clients living with HIV using implants for them to take along to their primary healthcare providers, stating they could retain the method if they chose to*, ‘so they can carry it with them, because they go to the clinic and clinic says, ‘No, you must have that out!’* (specialist contraception provider, female).

### Influence of training, capacity and health systems issues on implementation of implant-efavirenz guidance

Some providers and stakeholders observed that public sector healthcare providers had limited time to provide comprehensive client counselling and choice of contraceptive methods. This was considered especially true for LARCs, as well as for the implant in the context of efavirenz and other enzyme inducing drugs. Hence, the default position was to not counsel on or offer such methods. One provider described an integrated service environment where *‘the family planning sister must do everything. It’s more like a one stop shop that we’re currently busy doing. So, there are some services that you are going to neglect. When you’re there, and I must put in an [implant], and I know there’s a sick child – I must still see the sick children. I’m not going to put in the [implant]. I’m going to opt for something else. It won’t be the client’s choice, it will be the provider’s choice, because of the clients that must still be seen’* (professional nurse, female). An OB/GYN was also concerned that providers were often overburdened by the responsibility to *‘understand every single detail about every single condition that they’re dealing with’* (OB/GYN, male). He believed it was ‘*impossible*’ to expect providers to properly convey all necessary information to clients during single consultations and still find time to facilitate informed contraceptive choice, leading providers to focus on what they considered to be the more familiar methods which require less nuanced counselling.

Some stakeholders corroborated this, noting that, particularly in areas where there was limited provider training, they had observed a pattern of simplified rule-following amongst primary healthcare providers. This was understood as a way for providers to retain their ability to make efficient decisions whilst managing heavy workloads and wide-ranging responsibilities: ‘*So, a lot of the clinicians are doing things that they aren’t necessarily fully trained and equipped for, particularly the nurse clinicians, and so, they adopt one rule, and that’s just the way it happens*’ (pharmacologist, female). In the specific case of the implant-efavirenz guidance, *‘I do think it’s probably easier just to take out the implant – you just learn the rule and then you follow it’* (HIV clinician, female).

Notably, some stakeholders believed that inadequate training on implant provision and counselling (and subsequent confusion about implant-efavirenz guidance) affected both doctors and nurses in the public healthcare sector. ‘*There was an assumption that doctors don’t need [implant] training’* (health manager, female) and, when it came to incorrect insertions, ‘*doctors are worse than nurses’* (specialist contraception provider, female). HIV clinicians were believed to be more likely to misinterpret the NDoH advice *‘because a lot of the people who get referred to the family planning clinic to take the implant out come from the HIV clinics’* (OB/GYN, female).

### Suggestions for future contraceptive policy-making and service delivery initiatives

In light of their experiences around implant introduction and guidance on implant use for women living with HIV, participants were asked to suggest recommendations for improving contraceptive choice and services for women living with HIV in South Africa. Themes emerged related to: multi-stakeholder consultation when developing guidance; ensuring that contraceptive care is focussed on quality of counselling and services, which build client-provider trust, as opposed to numerical targets; and the NDoH undertaking efforts to improve the reputation of the implant specifically.

At the guidance and policy-making level, stakeholders emphasized that, when issuing guidance to providers, NDoH needed to take more time to consult with stakeholder groups, particularly in circumstances where available evidence indicates no immediate physical danger associated with an intervention or service. One health manager proposed a system of policy ‘*panel-beating’*. She explained that policy-makers *‘would improve a lot if they had things read and interpreted by somebody else. Sometimes, I get something, and I see it one way, and then somebody else reads it another way… So, I frequently call on the secretary, the cleaner, the clerk, and say, ‘Okay, read this for me. What did you understand?’* (health manager, female). Several stakeholders stated that significant gaps in primary healthcare provider knowledge about contraception generally, and the implant specifically, necessitated widespread retraining. This was due to the widespread non-provision of implants to all women living with HIV based on unfounded safety concerns, and regardless of ART regimen. It was viewed as urgent and timely given the anticipated upcoming changes in the first-line ART regimen in South Africa to a dolutegravir-based regimen (an ART considered highly unlikely to decrease the effectiveness of implants).

Provider and stakeholders suggested that when new contraceptive methods are introduced, implementation should focus more on ensuring that providers are equipped to facilitate client-centred, rights-based, informed contraceptive choice, rather than placing pressure on clinics to meet numerical targets for method coverage. Participants in both groups expressed frustration that ‘*pushing for numbers’* (professional nurse, female) in the case of the implant rollout had compromised client method choice. Related to this, emphasis in training is needed on building provider-client trust in contraceptive care and facilitating choice, especially in offering long-acting options such as the implant. As one provider explained: *‘We [providers] have this way of, you know, subtly forcing them to take something and, ultimately, they walk out and they’re angry with you as a nurse, as a facility. They’re angry because you’ve given them something that they didn’t want, and a three-year device is… It’s a three-year device. So, irrespective of if they have HIV or they don’t have HIV, I think if the client has the knowledge and consent – we need to treat clients as if they were adults’* (professional nurse, female).

Providers and stakeholders identified a need for the NDoH to actively address the implant’s broader negative reputation and misinformation about the implant amongst providers, women and communities. With regard to the implant-efavirenz directives in particular, providers stated that they need clarification about its safety and effectiveness in women living with HIV, and the rights of women living with HIV to choose the method. A health manager suggested engaging HIV activist groups, who *‘play a very important role in whether a method or system, or drug, or whatever, is going to be adopted or not. They are very strong activists, and most of them were scared that they had an [implant] inserted and they were on ARVs. What do you think they are going to tell the rest of the community?’* (health manager, female).

## Discussion

This study offers the first direct insight into the primary healthcare level impacts of the South African NDoH’s guidance on provision of implants to women living with HIV on efavirenz-based ART regimens. Discussions with providers revealed that, at all four clinics visited, implants were not currently offered to women living with HIV. This corresponded with stakeholders’ wider impressions of how the directives have been interpreted and implemented in primary healthcare settings across Cape Town. Based on this guidance, providers and clinics were effectively denying women living with HIV access to the contraceptive implant.

Our data suggests that several composite factors may have led to primary healthcare providers implementing the implant-efavirenz guidance in this very restrictive way. Stakeholders recalled that the initial implant provider training programme and rollout in 2014 had been rushed and poorly coordinated, and felt providers were not appropriately equipped to counsel women about the new method in general and less so about more nuanced and more individualised issues related to implant use among women living with HIV. Stakeholder and provider perceptions on the South African implant introduction and early client and provider concerns about the method that emerged in our study are supported by other South African research on this topic [16, 18, 29]. By the time the implant-efavirenz guidance was issued, providers had thus already developed scepticism about the suitability of the method for their clients in general. The directives’ wording was perceived by participants to imply the possibility of harm to a woman’s health associated with the implant’s interaction with efavirenz, rather than solely reduced contraceptive effectiveness. Several participants expressed doubt that many providers, under pressure to provide integrated services in busy public sector clinical environments, could have realistically achieved a nuanced understanding of the directives, given that these updates were just two among many they were expected to interpret and implement. Providers tended to adopt the ‘safe and simple’ rule that implants were contraindicated with HIV infection, regardless of ART regimen.

Lipsky’s ‘street-level bureaucracy’ theory suggests that professional discretion used by civil servants who interact directly with members of the public often fundamentally shapes the way policies associated with public services are implemented [30]. In the presence of structural pressures such as heavy workloads, time constraints, resource limitations, and training deficiencies, the capacity of individuals working on the ground to interpret and implement incoming policies and updates may be compromised [31]. This predicament tends to result in the street-level bureaucrat, in this case the primary-level healthcare provider, implementing informal, often simplified versions of policies as a ‘coping strategy’. Such coping behaviour may have been particularly pronounced in the case of providers implementing the implant-efavirenz directives in this study, where it was perceived that a ‘judgement call’ to offer or provide the implant to a woman living with HIV may have risked harm to a client, or resulted in legal ramifications for the provider.

This study has implications for implant-efavirenz directive implementation by primary providers elsewhere in South Africa. If representative of how the implant-efavirenz directives have been implemented by primary providers elsewhere, the findings of this study are concerning. They suggest that South African women living with HIV are potentially being denied the option to use a safe, convenient and effective contraceptive method. This is a contravention of women’s sexual and reproductive health rights [32–34] and contradicts the intentions of the revisions made to the South African contraception guidelines in 2012, which sought to expand contraceptive choice for all women, including women living with HIV [3, 12, 13]. It additionally contradicts WHO MEC guidance on implant provision in the context of efavirenz-based ART [14]. With impending changes to South Africa’s first-line ART regimen (in the near future, efavirenz will be replaced by dolutegravir [35, 36], which is a differently classed drug to efavirenz and one that is unlikely to cause similar drug-drug interactions with the contraceptive implant), it is important that the South African policy environment reconsiders the available evidence, that guidance is updated and clearly communicated, and that providers are retrained. Providers need support through training and tools to better understand and accurately deliver the important counselling message of reduced implant effectiveness with efavirenz, while still allowing efavirenz-using clients to choose the implant. They also need training to understand and communicate that as dolutegravir, which is unlikely to reduce implant effectiveness, replaces efavirenz, and counselling messages on this topic will need to change.

Participants’ suggestions for improving the quality of contraceptive care to women were similar to existing literature [3, 16, 18]. In policy-making, sufficient time should be taken to conduct an appraisal of evidence and stakeholder perspectives to minimize unintended consequences and misinterpretation of recommendations. Provider retraining on implant provision and counselling is needed, as well as on choice-based, client-centred contraceptive care. Counselling could be made more time-efficient in busy clinical settings with standardized messages and decision-making tools and user-friendly materials for clients to absorb in clinic waiting rooms and at home. Targeted provider and community engagement to improve perceptions of and dispel misinformation about the implant generally is needed.

### Limitations

This qualitative study of 20 participants is limited by being confined to the Cape Town Metropolitan Area. It may therefore not be generalizable. However, the insights gained are likely to have transferability to other public sector primary healthcare settings in South Africa. The participating clinics serve a relatively broad cross-section of communities using public sector primary healthcare services. The women utilizing contraceptive services at the clinics were from diverse South African and migrant backgrounds and of various reproductive ages, including adolescents and young adults. The clinics were also situated in a variety of low-income urban and peri-urban areas. In addition, stakeholders were chosen to represent a diversity of expertise and experience pertaining to HIV and/or contraceptive care and service delivery. Interviews being conducted in English may have resulted in some limitations; however, all participants indicated comfort with English for interviews and this is the usual language used in the healthcare workspace in South Africa. Power imbalances between the interviewer and participants may have existed; however, in qualitative research, the researcher is deeply cognisant of power relations and triangulation of data was used, with the aim of minimizing the impact of power imbalances on data quality.

## Conclusions

The findings of this study suggest that the South African NDoH’s 2014 guidance on contraceptive implant use among women using efavirenz may have had unintended consequences at the primary care level. It may have effectively removed the safe, effective and convenient implant as a contraceptive option for all women living with HIV, thus, denying them informed contraceptive choice and infringing on their sexual and reproductive health rights. The appropriateness of the recommendation against implant use in the context of efavirenz-based ART is still under debate. The upcoming move to dolutegravir-based ART in South Africa and elsewhere underscores the urgent need for further investigation and evidence-based action by international bodies, countries and HIV and family planning programmes. A challenge for future research and policy development in this area will be creating and implementing robust and effective training and re-training programmes and counselling tools that enable and entrench the principal of individualised, informed contraceptive choice for clients. This is particularly important for providers working in busy multipurpose integrated health service environments. Service beneficiaries and stakeholders at all levels should be consulted throughout policy development, decision-making and introduction phases, so that guidance stands the best chance of being correctly interpreted and well-implemented. The study additionally provides some insights and lessons for introduction of other new public health products.

## Data Availability

The datasets used and/or analyzed during the current study are available from the corresponding authors upon reasonable request. No identifiable information about participants will be distributed.

## Abbreviations

ART: Antiretroviral therapy
HIV: Human immunodeficiency virus
MEC: Medical Eligibility Criteria
NDoH: National Department of Health
OB/GYN: Obstetrician/Gynaecologist
WHO: World Health Organization
